# Virtualizing the p-ANAPL Library: A Step towards Drug Discovery from African Medicinal Plants

**DOI:** 10.1371/journal.pone.0090655

**Published:** 2014-03-05

**Authors:** Fidele Ntie-Kang, Pascal Amoa Onguéné, Ghislain W. Fotso, Kerstin Andrae-Marobela, Merhatibeb Bezabih, Jean Claude Ndom, Bonaventure T. Ngadjui, Abiodun O. Ogundaini, Berhanu M. Abegaz, Luc Mbaze Meva’a

**Affiliations:** 1 Department of Chemistry, Chemical and Bioactivity Information Centre, Faculty of Science, University of Buea, Buea, Cameroon; 2 Department of Chemistry, Faculty of Science, University of Douala, Douala, Cameroon; 3 Department of Organic Chemistry, University of Yaoundé I, Yaoundé, Cameroon; 4 Department of Biological Sciences, University of Botswana, Gaborone, Botswana; 5 Department of Chemistry, University of Botswana, Gaborone, Botswana; 6 Department of Pharmaceutical Sciences and Traditional Pharmacopeia, Faculty of Medicine and Biomedical Sciences, University of Yaoundé I, Yaoundé, Cameroon; 7 Department of Pharmaceutical Chemistry, Obafemi Awolowo University, Ile-Ife, Nigeria; 8 Academy of Sciences, Nairobi, Kenya; Texas Tech Univ School of Pharmacy, United States of America

## Abstract

**Background:**

Natural products play a key role in drug discovery programs, both serving as drugs and as templates for the synthesis of drugs, even though the quantities and availabilities of samples for screening are often limitted.

**Experimental approach:**

A current collection of physical samples of > 500 compound derived from African medicinal plants aimed at screening for drug discovery has been made by donations from several researchers from across the continent to be directly available for drug discovery programs. A virtual library of 3D structures of compounds has been generated and Lipinski’s “Rule of Five” has been used to evaluate likely oral availability of the samples.

**Results:**

A majority of the compound samples are made of flavonoids and about two thirds (2/3) are compliant to the “Rule of Five”. The pharmacological profiles of thirty six (36) selected compounds in the collection have been discussed.

**Conclusions and implications:**

The p-ANAPL library is the largest physical collection of natural products derived from African medicinal plants directly available for screening purposes. The virtual library is also available and could be employed in virtual screening campaigns.

## Introduction

The African continent is highly endowed with diverse vegetation types constituting tropical rainforests, coastal and alpine forests, savannahs, woodlands and scrublands which is a reservoir for diverse natural product (NP) classes [1], [2]. Natural products have always served as sources of inspiration for the design of new drugs and/or as drugs themselves [3]–[5]. In addition, it has been verified that natural products from Africa have an enormous potential for drugs [6]. The measured activities of compounds derived from plants of African origin have been reported in at least 2000 publications and it is believed that the active principles for the development of drugs against some of the neglected tropical diseases may be found in the African flora [5], [6], since some of these plants have been used extensively in the treatment of some of these diseases in African Traditional Medicine (ATM) [7]. In addition, the potential of natural products derived from African medicinal plants has been demonstrated by a number of recent review articles [6], [8]–[16]. In order to properly exploit these data for drug development, African researchers have initiated efforts towards the collection of compound samples which should be readily available for screening. It is important to note that although natural products play important/evolving roles in drug discovery [17], huge pharmaceutical companies have reduced emphasis on natural products in terms of new drug development for the last two decades [17]–[19]. There are many reasons for this decreasing interest is the strategy of drug discovery employed by pharmaceutical companies, of which the most important is the time factor involved in the search for NP lead compounds. The trend has been drastically changing from a laborious search for potent NP leads (bioassay-guided isolation of natural products from crude extracts, without guaranteeing reasonable quantities of isolates for testing) to the rapid high-throughput screening of molecular target-based pure compound chemical libraries. The chemical libraries are often generated, to a large extent, using combinatorial chemistry. However, streamlined screening procedures and enhanced organism sourcing mechanisms are among the new technologies which have been put in place in order to enhance natural product drug discovery in an industrial setting [20]. In order to considerably reduce the number of compound samples required for testing in bio-assays in drug discovery projects, computer-aided methods like docking and virtual screening (VS) have been employed. Computer-aided drug design (CADD) often involves VS of large compound datasets and the availability of such is vital for drug discovery protocols. Thus, the development of virtual libraries for this purpose is of utmost importance in a modern drug discovery program [21]–[23]. In this paper, we demonstrate that a “drug-like” and diverse library, constituting > 500 natural products collected from diverse regions within the continent of Africa (for which samples are directly available for screening), might be both a significant starting point and a useful tool in the drug discovery process, if virtual and biological screenings are employed.

What is p-ANAPL? The pan-African natural products library (p-ANAPL) is a consortium of natural product collections isolated from African biota and owned by scientists and/or groups of scientists working in African institutions. The p-ANAPL project was established in April 2009 by a group of natural product scientists from Botswana, Cameroon, Ethiopia, Kenya, Sudan and Tanzania, to build up the collection of natural products and to create an enhanced and efficient platform for their biological screening, and thereby promote the discovery of useful products from them. The p-ANAPL library is associated with the Center for Scientific Research, Indigenous Knowledge and Innovation (Cesriki), in the University of Botswana.

## Materials and Methods

### Collection method of the physical library

The compounds in the p-ANAPL collection were mainly obtained through the Network for Analytical and Bioassay Services in Africa (NABSA) which is based at the Department of Chemistry at the University of Botswana. NABSA was initiated in 1992 to share facilities with other African scientist working in facility-constrained environments. NABSA promotes and continues to do so, short-term visits by African researchers and postgraduate students to the University of Botswana and providing access to spectroscopic services (High Field NMR, High Resolution MS, Chiroptical Spectroscopy) for less privileged researchers and institutions. NABSA therefore promoted intra-African cooperation amongst several countries including Cameroon, Nigeria, Kenya, Ethiopia, Tanzania, the Democratic Republic of Congo and Zimbabwe. It is the policy of NABSA that visiting researchers who isolate and elucidate the structures of natural products voluntarily deposit specimens to establish a collection of natural products with full entitlement to any intellectual property (IP) that may arise from subsequent assay of these substances. It is mostly these compounds that formed the initial basis for p-ANAPL.

The first p-ANAPL consortium meeting in Gaborone, Botswana in 2009, defined its main goal as systematically organising compounds in a well-characterized natural product collection with the potential to expand and to use for bioactivity screening purposes. One of the co-authors of this paper, G. W. F., undertook the task of characterization and cataloguing all compounds forming the basis of this paper during a post-doctoral term at the University of Botswana from 2011 to 2013. The challenges and lessons learnt from this exercise will be published elsewhere.

### Generation of 3D models and in silico determination of molecular descriptors

The 3D structures of the compounds had been sketched and energy minimisation subsequently using the graphical user interface (GUI) of the MOE software [24] running on a Linux workstation with a 3.5GHz Intel Core2 Duo processor. The 3D structures were generated using the builder module of MOE and energy minimization was subsequently carried out using the MMFF94 force field [25] until a gradient of 0.01 kcal/mol was reached. The 3D structures of the compounds were then saved as.mol2 files subsequently included into a MOE database (.mdb) file and converted to other file formats (.sdf,.mol,.mol2 and.ldb), which are suitable for use in several virtual screening workflow protocols. The molar weight (MW), number of rotatable bonds (NRB), lipophilicity parameter (log *P*), number of hydrogen bond acceptors (HBA), number of hydrogen bond donors (HBD) and number of Lipinski violations were calculated using the molecular descriptor calculator included in the QuSAR module of the MOE package [24]. A further treatment was carried out to ensure that the protanation states of the compounds were correct with respect to biological pH (strong acids were deprotonated, strong bases protonated and metal atoms removed). A maximum of 10 tautomers were generated for each molecule in the dataset.

## Results and Discussion

### Unique natural compounds within the library

The compounds in the p-ANAPL collection comprise about thirty (30) different classes of compounds that reflect a substantial chemical diversity even in a relatively small collection. It shows the potential of African natural products in terms of molecular diversity which has been noted to be often limited in large compound libraries used in high throughput screening (HTS) format [21], [26]. Molecular diversity goes hand in hand with bioactivity diversity, which is an important consideration for drug discovery. A recent study based on a database of 197 201 natural products (NP) from plants, animals and microorganisms of mainly Chinese origin revealed that there was a large overlap between natural products and FDA-approved drugs, in terms of chemical space, indicating the potential of NPs to become lead compounds [27]. An analysis of NP-target networks suggested a high degree of polypharmacology associated with NPs. This seems to confirm comparisons by Feher and Schmidt, showing that overall, NPs are more similar to drugs than compounds obtained from combinatorial chemistry [28]. Close to half of the p-ANAPL compounds are flavonoids (mono-, di-, tri- prenylated and geranylated flavones, flavanones, chalcones; homoisoflavonoids, rotenoids, biflavonoids, bichalcones, etc.), a very versatile class of NPs, which have been associated with a relatively large margin of safety for therapeutic use in humans and which have recently been reported to display diverse anti-HIV bioactivities [29]. More than two hundred (200) flavonoids, such as proropensin (**1**) or Dinklagin B (**2**), isolated primarily from *Dorstenia* species [30], [31], Figure 4, are available in good quantities in the p-ANAPL collection. The p-ANAPL collection, furthermore, contains about forty (40) homoisoflavonoids isolated mainly from Hyacinthaceae species such as *Scilla nervosa* [32], which constitutes a quite unique contingent and the largest collection of compounds representing this class. An initial assessment of commercial availability of p-ANAPL compounds from major suppliers, PubChem entries and specifically dedicated webpages for supplies of chemicals revealed that approximately half of the compounds are currently not commercially available. Amongst them are compounds with very unique properties. Examples are the phenyl anthraquinones, Gaboroquinone A and B (**3** and **4**), with interesting stereochemistry and good antiplasmodial (IC_50_ = 4.2 μg mL^−1^ against *Plasmodium falciparum* (NF54)) and anti-trypanosomal (IC_50_ = 5.1 μg mL^−1^ against *Trypanosoma b. rhodesiense*) activities [33].

Furthermore, the p-ANAPL collection contains a series of sulphate derivatives of 6′-*O*-sulphated phenylanthraquinones, which are unique as natural products with sulphate functional groups are considered to be very rare in plants [34]. The p-ANAPL also contains the largest collections of isofuranonapthoquinones. Broussoflavonol B (**5**), contained in the p-ANAPL collection, is a recently identified novel anti-cancer agent which has been shown to inhibit growth of estrogen-negative breast cancer MDA-MB-231 cells, a particularly more malignant and aggressive form of cancer cells which account for about one third of breast cancers [35]. Additionally, the collection contains julocrotine (**6**), an alkaloid, which has been shown to be effective against *Leishmania (L.) amazonensis amastigotes*, the causative agent for cutaneous leishmaniasis (IC_50_ = 19.8 μM) [36], and a series of compounds dorsmanin A to I, isolated from the genus *Dorstenia*, which showed versatile antimicrobial activities [37].

A substantial number of NP databases have been established in the last years [21], [38]–[43], which provided valuable information for assessments of chemical space occupation for virtual screening purposes. To a much lesser extent non-commercial actual NP collections with proper characterization and traceability of samples do exist for the African continent. An example of a similar effort is the Natural Product Discovery Institute (NPDI, formerly Merck U.S. Natural Products Library and Schering-Plough Legacy Culture Collection, www.npdi-us.org) and the Developmental Therapeutics Program (DTP) collection of the National Cancer Institute/National Institutes of Health (NCI/NIH) in the US (http://dtp.nci.nih.gov/branches/npb/repository.html), with only a partial coverage of African natural resources. Both collections are not based on the African continent. Close to half of the p-ANAPL compounds are available in amounts of more than 10 mg which allows biological screening to some extent. Therefore, the p-ANAPL collection is unique in the sense that it has brought together NPs from African researchers that remain under the control of African institutions. We hope that this first virtual characterization of our collection triggers interest in that collection and, above all, serves as a springboard to extend p-ANAPL to other African research institutions to transform it into a true pan-African collection.

### The chemical composition of the p-ANAPL library

**Figure 1:**
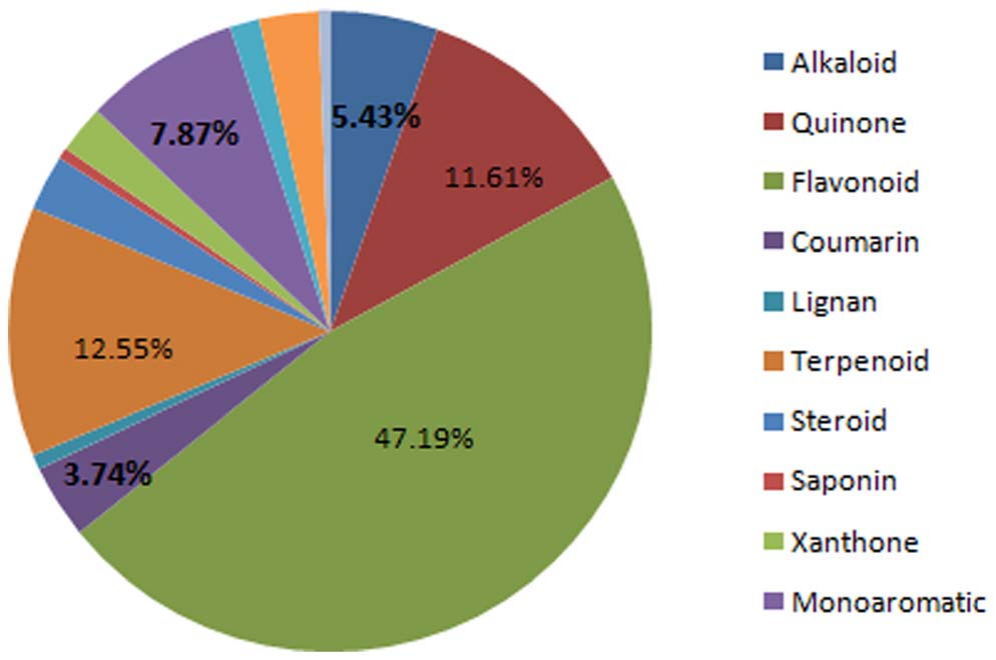
Pie chart showing the distribution by compound type.

A pie chart, showing the distribution of the five hundred and thirty four (534) samples currently lodged in the p-ANAPL library is shown in Fig. 1. As previously mentioned, a majority of the compound samples are flavonoids (flavanones, biflavonoid, isoflavones, homoisoflavonoids, and chalcones), constituting 47.19% of compound samples. This is followed by terpenoids (12.54%), quinones (11.61%), monoaromatic compounds (7.87%), alkaloids (5.43%), coumarins (3.74%), steroids (2.81%) and xanthones (2.43%). The remaining compound types each represented < 2% of the total sample collection.

### Assessment of its “drug-likeness” and “lead-likeness” potential

**Figure 2:**
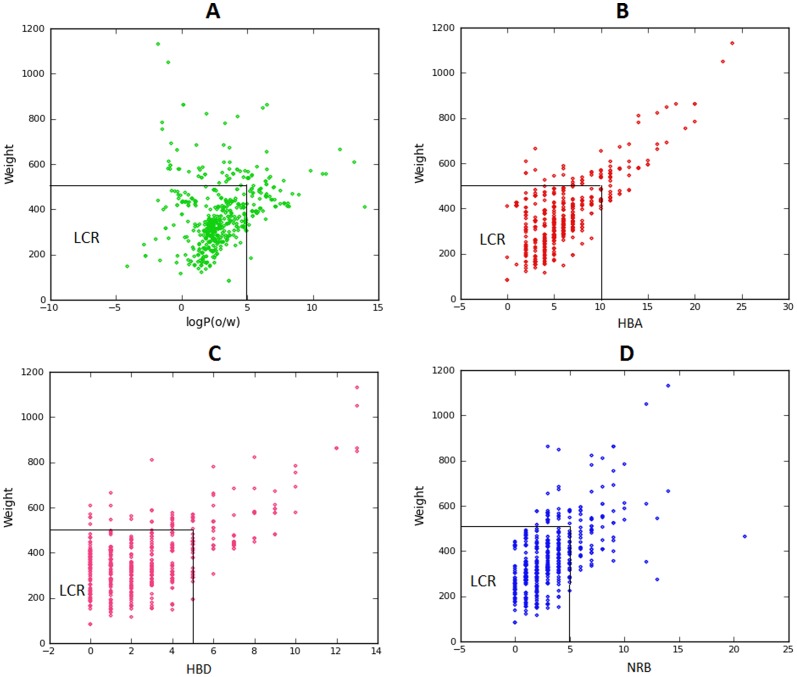
Pair-wise distribution of Lipinski parameters for the p-ANAPL library; (A) log P against MW, (B) HBA against MW, (C) HBD against MW and (D) NRB against MW.

Lipinski’s “Rule of Five” [44] (ro5) is a very useful tool for assessing chemical compound libraries to be used in drug discovery programs. This “rule” was derived from chemical libraries from the World Dug Index (WDI), as a criterion to evaluate likely oral bioavailability [44]–[45]. However, the highly valuable class of NPs was initially omitted, since Lipinski had postulated that the ro5 was not respected by NPs. NP libraries have however been previously analysed comparatively using the ro5 in order to have a rough idea of the extent of “drug-likeness” of a compound library to be used in virtual screening [21], [41]–[43]. It is on these grounds that Lipinski’s criteria [44] are often used for the evaluation of “drug-likeness” of compounds within the designed libraries. Thus, Lipinski’s ro5 is often regarded as a useful filter for the elimination of compounds not likely to be orally available in the early stages of drug discovery protocols [45]. In summary, Lipinski’s ro5 defines a “drug-like” molecule as one with high likelihood to be orally available, for which the molar weight (MW) ≤ 500 Daltons (Da), the logarithm of the octan-1-ol/water partition coefficient (log P_(o/w)_ or log P) ≤ 5, the number of hydrogen bond acceptors (HBA) ≤ 10 and the number of hydrogen bond donors (HBD) ≤ 5. An additional rule for the number of rotatable bonds (NRB ≤ 5) is often included to the ro5. An evaluation of “lead-likeness” is often carried out using more stringent criteria defined by Oprea *et al* [46]–[49]. The “Rule of 3.5” for “lead-like” compounds is defined as: 150 ≤ MW ≤ 350; log P_(o/w)_ ≤ 4; HBD ≤ 3; HBA ≤ 6). The criteria for “fragment-like” libraries is defined by Verdonk *et al* [50]. The criteria (also referred to as the “Rule of 2.5”) are such that MW ≤ 250; –2 ≤ log P_(o/w)_ ≤ 3; HBD < 3; HBA < 6; NRB < 3. A pairwaise scatter plot for the physico-chemical parameters defining the ro5 are shown in Fig. 2. These plots show that the regions of highest population density of points fall within the Lipinski compliance regions (LCR).

**Figure 3:**
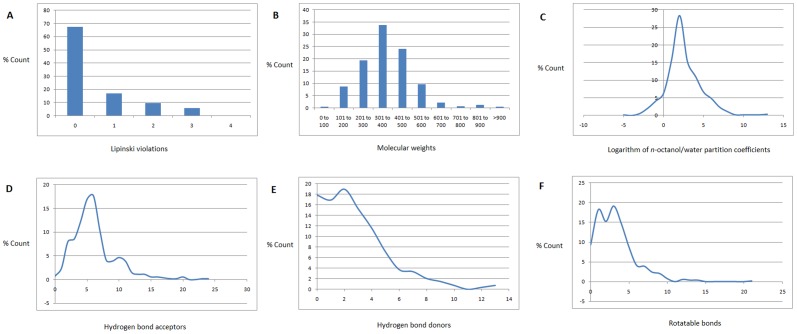
Distribution of Lipinski parameters for the p-ANAPL library; (A) Bar chart showing the number of Lipinski violations, (B) Bar chart showing the MW parameter, (C) Plot of the lipophilicity parameter, (D) Plot of the HBA parameter, (E) Plot of the HBD parameter, and (F) Plot of the NRB parameter.

Fig. 3 shows the distribution of violations and compliance of Lipinski parameters in the p-ANAPL library, while the maxima, minima and mean values for each parameter are shown in Table 1, along with those of the “drug-like”, “lead-like” and “fragment-like” subsets, defined following the aforementioned criteria. An analysis of the total library showed that about 67% of the compounds had no Lipinski violations and almost 85% of the compounds showed < 2 violations (Fig 3A). The ‘drug-likeness’ is further highlighted by an analysis of individual parameters (MW, log P_(o/w)_, HBA and HBD). The MW values showed a Gaussian distribution with a peak value located between 301 and 400 Da, Fig. 3B. This interval corresponded to about 34% of the compounds currently included in the p-ANAPL database. In addition, only about 14% of all the compounds showed MW falling outside the recommended range for the ro5 (> 500 Da). The mean values for MW ( =  370 Da), further highlighted the “drug-like” nature of our library, the mean MW of a typical drug being equal to 310 Da [51]. The log P_(o/w)_ distribution curve (Fig. 3C) showed a very steep Gaussian shape with a peak centred at 2.5 log P_(o/w)_ units, with only 9.5% of the compounds having log P_(o/w)_ > 5. Only six compounds (compounds **8** to **13**) showed log P_(o/w)_ > 8 units. These constitute stilbenoid and long chain alkyl esters of some pentacyclic triterpenoids (Fig. 5). The distribution curves for HBA and HBD respectively rose rapidly to maxima of 6 acceptors (corresponding to 17.66% of the compounds) and 2 donors (corresponding to 18.96% of the compounds). The two graphs then fell to 24 acceptors and 13 donors respectively (Fig. 3D,E). Moreover, only 10.41% of the compounds had HBA > 10 and only 12.45% of the compounds showed HBD > 5. The graph of the NRB showed two cusps; at 1 and 3 RBs (Fig. 3F) and rapidly fell to 21 rotatable bond (RBs), with only 14.68% of the compounds having NRB > 5. The mean values of all the Lipinski parameters indicate a high probability of finding ‘drug-like’ and ‘lead-like’ compounds within the p-ANAPL library (Table 1).

**Figure 4:**
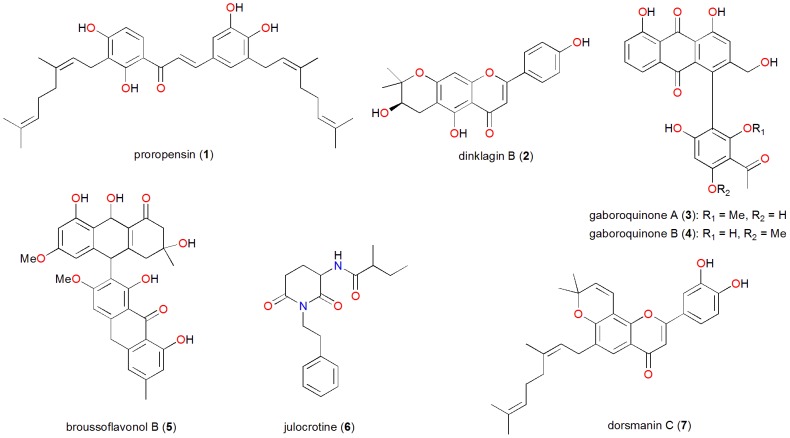
Chemical structures of selected bioactive natural products from the p-ANAPL library (1 to 7).

**Figure 5:**
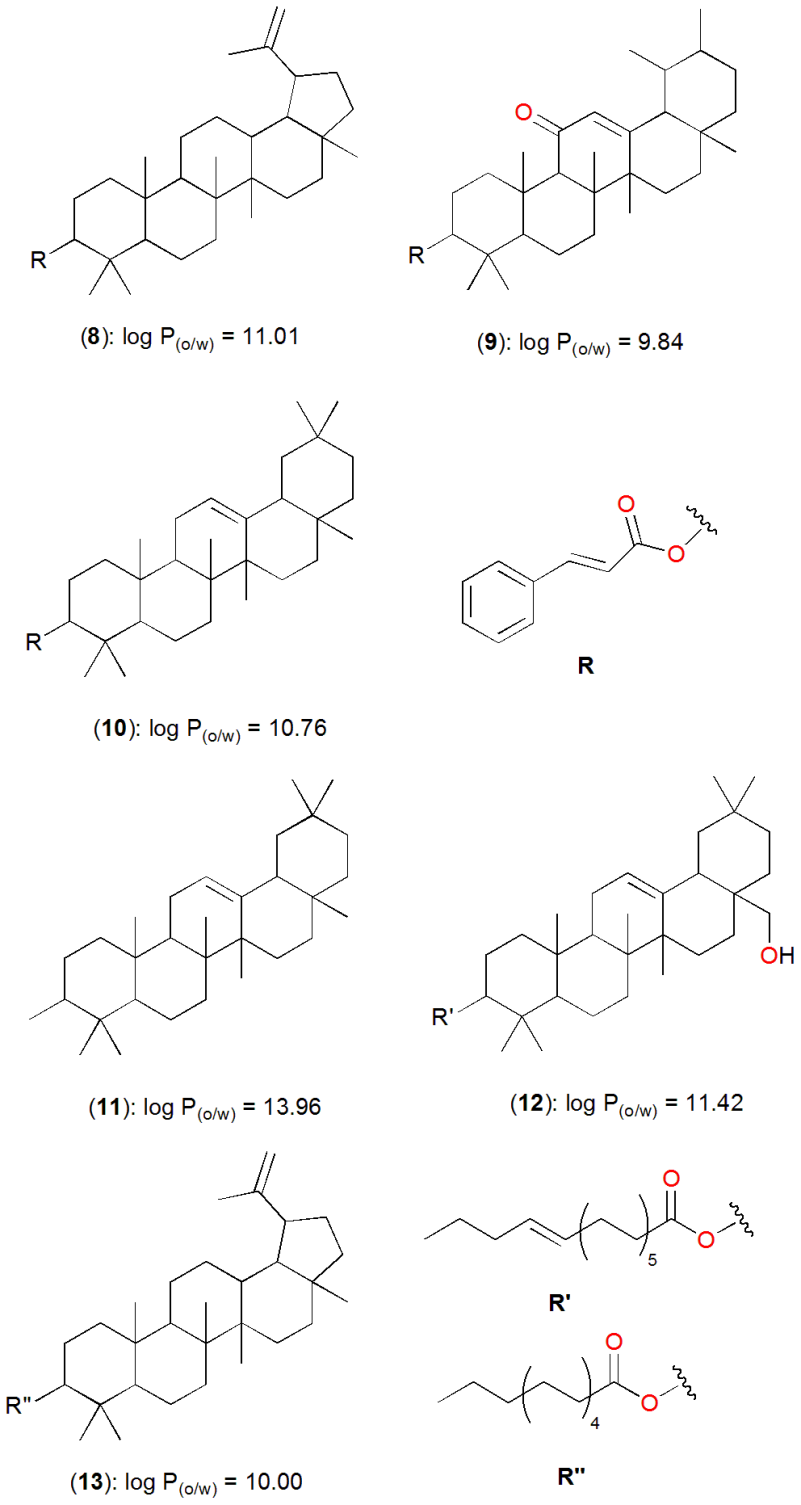
Chemical structures of natural products from the p-ANAPL library with log P > 8 units.

**Table 1:** Summary of physico-chemical properties (often used to predict ‘drug-likeness’) of the compounds within the p-ANAPL library.

aLib. name	aLib. size	*^b^*Taut.	*^c^*MW (Da)			*d*l°gP_(o/w)_			*^e^*HBA			*^f^*HBD			*^g^*NRB		
			*^h^*Max.	*^i^*Min.	*^j^*Avg.	*^h^*Max.	*^i^*Min.	*^j^*Avg.	*^h^*Max.	*^i^*Min.	*^j^*Avg.	*^h^*Max.	*^i^*Min.	*^j^*Avg.	*^h^*Max.	*^i^*Min.	*^j^*Avg.
p-ANAPL	534	1428	1131.27	84.16	370.19	13.96	–4.12	3.02	24	0	6.12	13	0	2.79	21	0	3.26
*^k^*Drug-like	363	943	487.55	84.16	303.22	5.98	–4.12	2.48	10	0	5.26	5	0	2.07	13	0	2.57
*^l^*Lead-like	304	774	448.43	84.16	285.49	5.30	–4.12	2.42	8	0	4.98	5	0	2.03	7	0	2.37
*^m^*Fragment-like	61	115	248.32	84.14	190.08	3.62	–0.36	1.76	5	0	3.20	3	0	1.16	2	0	0.98

a Library; *^b^*Number of tautomers; *^c^*Molecular weight; *^d^*Logarithm of *n*-octanol/water partition coefficient; *^e^*Number of hydrogen bond acceptors; *^f^*Number of hydrogen bond donors; *^g^*Number of rotatable single bonds; *^h^*Maximum number; *^i^*Minimum number; *^j^*Mean value; *^k^*The “drug-like” library was selected following Lipinski’s criteria [44]–[45]; *^l^*The “lead-like” library was selected following Oprea’s criteria [46]–[48]; *^m^*The “fragment-like” library was selected following Verdonk’s criteria [50].

### A discussion of pharmacological profiles of selected compounds

**Figure 6:**
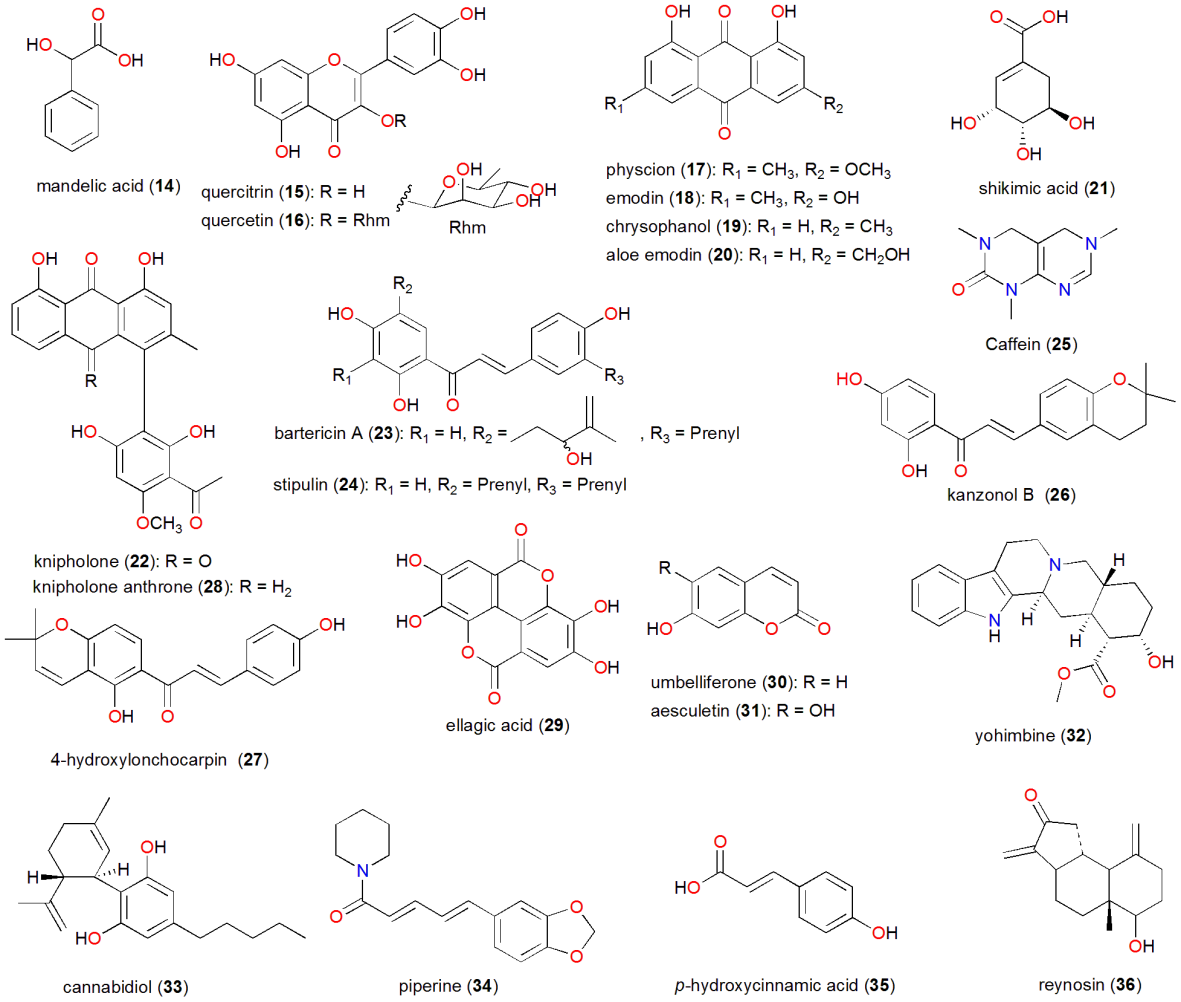
Chemical structures of selected bioactive natural products from the p-ANAPL library (14 to 36).

The chemical structures of selected compounds with interesting pharmacological profiles from the p-ANAPL library have been shown in Fig. 6. Esters of the monoaromatic mandelic acid (**14**) have been involved in the modulation of the enantioselectivity of lipases via controlled immobilization on glutaraldehyde supports [52]. In addition to previously known biological activities, Zofou *et al*. recently demonstrated the anti-malarial properties of the flavonoids quercitrin (**15**) and quercetin (**16**), derived from *Dacryodes edulis* (Burseraceae), a plant currently used in the treatment of malaria and fevers in West Cameroon [53]. Compound **15** exhibited IC_50_ values of 5.96 and 2.26 μg mL^−1^, against the 3D7 and Dd2 strains of *Plasmodium falciparum* respectively, while compound **16** exhibited IC_50_ values of 6.07 and 5.91 μg mL^−1^, against 3D7 and Dd2 respectively. The anthraquinones emodin (**18**) and aloe emodin (**20**) have demonstrated interesting anticancer properties [54]–[57], while chrysophanol (**19**) has shown the ability to induce necrosis through the production of ROS and alteration of ATP levels in J5 human liver cancer cells [58], in addition to its antidiabetic [59], anti-inflammatory [60] and antimicrobial [61]–[62] properties. Moreover, chrysophanol-8-*O*-glucoside, has shown antiplatelet and anticoagulant activities [63]. It is also proven that physcion (**17**) and emodin (**18**) exhibit antibacterial properties against three *Bacillus* species, emodin exhibiting MICs in the range 0.5 – 2.0 μg mL^−1^ [64]. Both compounds inhibited *Pseudomonas aeruginosa*, emodin being more effective, showing an MIC of 70 μg mL^−1^ [64].

Shikimic acid (**21**) is known to play a key role in aromatic biosynthesis [65]. This has been exploited in the design of modified shikimic acid derivatives as potential anti-tubercular agents, inhibiting *Mycobacterium tuberculosis* shikimate kinase enzyme [66]. The quinone knipholone (**22**) has been derived from *Kniphophia foliosa* (Asphodelaceae) and is known to exhibit anti-malarial activity [67]. The antiplasmodial activity of knipholone is IC_50_ = 0.38 μM compared to Chloroquine 0.09 μM [68]. The chalcones bartericin A (**23**), stipulin (**24**), kanzonol B (**26**) and 4-hydroxylonchocarpin (**27**) have also shown anti-malarial activities [69]. while caffein (**25**) is a common psychostimulant. Caffeine was also reported to inhibit HIV-1 transduction of non-dividing cells [70]. Knipholone anthrone (**28**) has shown anti-cancer activity, with IC_50_ of 0.5 to 3.3 μM against leukaemic and melanocyte cancer cell lines [71]. Ellagic acid (**29**), isolated from the leaves of *Alchornea cordifolia* (Euphorbiaceae) by Banzouzi *et al*. [72], also showed good activity against *Plasmodium berghei* in mice with an ED_50_ in the range of 0.2 – 0.151 μg mL^−1^. The coumarin umbelliferone (**30**) has exhibited analgesic and anti-inflammatory [73], antihyperglycemic [74] and antioxidant [75]–[76] activities. The derivatives of umbelliferone have proven to be potent 5α-reductase type 1 inhibitors and provided the basis for further development for the treatment of human endocrine disorders associated with overproduction of DHT by 5α-reductase type 1 [77]. Aesculetin (**31**) induces apoptosis through a ROS-mediated mitochondrial dysfunction pathway in human cervical cancer cells [78], in addition to its antimicrobial [79], and anti-inflammatory [80] activities.

Yohimbine (**32**) is an alkaloid extracted from *Pausinystalia johimbe*, a plant commonly used to treat erectile dysfunction in ATM in West and Central Africa [2]. Both yohimbine [81] and its hydrochloride [82] have proven to be potent in the treatment of erectile dysfunction by preferential blockade of presynaptic α-adrenoceptors in rabbits [83]. Starke *et al*. demonstrated that yohimbine is more potent in blocking the presynaptic than the postsynaptic α-adrenoceptors of the artery. Cannabidiol (**33**) is a non-psychotropic component of *Cannabis* with possible therapeutic use as an anti-inflammatory drug [84]. It is a neuroprotective antioxidant [85], an oral anti-arthritic therapeutic in murine collagen-induced arthritis [86], which induces anxiety and psychotic-like symptoms in healthy subjects [87]. The plant alkaloid piperine (**34**) exhibits several biological activities, including inhibition of ethidium bromide efflux in *Mycobacterium smegmatis* [88], inhibition of Rv1258c, a putative multidrug efflux pump of *Mycobacterium tuberculosis* [89], selective inhibition of CYP3A4 [90], immunomodulatory and antitumor activities [91], inhibition of human P-glycoprotein [92], anti-inflammatory and antiarthritic effects [93]–[94], and inhibition of mammosphere formation [95]. Compound **35** (*p*-hydroxycinnamic acid) is known to exhibit anti-malarial activity [96], stimulate bone formation/inhibit bone resorption [97] and act as a natural mediator for laccase oxidation of recalcitrant compounds [98]. Reynosin (**36**) has exhibited neuronal toxicity protection in Parkinson’s disease models [99].

### Accessibility of the compounds

The virtual compound library is available as supplementary data accompanying this publication (Dataset S1). Updated versions of this dataset will be subsequently be available on request for non-commercial purposes through the corresponding author L. M. M. of this article. The 3D structures have been generated based on the chemical structure reported in the literature, and treated as previously described [41]–[43]. All requests for compounds samples for biological assays should be formally addressed to the corresponding author K. A. M. of this article.

## Conclusions

We have recently reported the development of natural product virtual libraries for African medicinal plants [41]–[43]. However, to the best of our knowledge, the p-ANAPL library constitutes the largest collection of physical samples of NPs derived from African medicinal plants, which is available for biological screening. In addition, the virtual library is provided in several file formats (mol2, sdf, mdb, and ldb), which are readable by several drug discovery software. These could be useful in virtual screening campaigns. Concerning the physical samples, the purity of each compound was tested by measurement of melting points, and confirmed to be > 95% pure before including in the database. The stability has been assured by keeping compounds at below 0°C in freezers. The uniqueness of the p-ANAPL library also lies in the fact that it is the largest collection of NPs with physical samples, specifically derived from African medicinal plants.

## Supporting Information

Dataset S1 **Electronic Supplementary Information (ESI) available: [Low energy 3D structures of compounds currently included in the p-ANAPL library for virtual screening, along with the generated tautomers, drug-like, lead-like and fragment-like subsets].** Correspondence and requests for materials should be addressed to LMM and KAM for the virtual library and physical samples respectively.
(RAR)
